# Control of assembly of extra-axonemal structures: the paraflagellar rod of trypanosomes

**DOI:** 10.1242/jcs.242271

**Published:** 2020-05-27

**Authors:** Aline A. Alves, Heloisa B. Gabriel, Maria J. R. Bezerra, Wanderley de Souza, Sue Vaughan, Narcisa L. Cunha-e-Silva, Jack D. Sunter

**Affiliations:** 1 Department of Biological and Medical Sciences, Oxford Brookes University, Oxford OX3 0BP, UK; 2 Instituto de Biofísica Carlos Chagas Filho, Universidade Federal do Rio de Janeiro, Rio de Janeiro 21941-902, Brazil; 3 Centro Nacional de Biologia Estrutural e Bioimagem (CENABIO), Universidade Federal do Rio de Janeiro, Rio de Janeiro 21941-902, Brazil

**Keywords:** Extra-axonemal, Flagellum, Assembly, Trypanosomes

## Abstract

Eukaryotic flagella are complex microtubule-based organelles that, in many organisms, contain extra-axonemal structures, such as the outer dense fibres of mammalian sperm and the paraflagellar rod (PFR) of trypanosomes. Flagellum assembly is a complex process occurring across three main compartments, the cytoplasm, the transition zone and the flagellum itself. The process begins with the translation of protein components followed by their sorting and trafficking into the flagellum, transport to the assembly site and incorporation. Flagella are formed from over 500 proteins and the principles governing assembly of the axonemal components are relatively clear. However, the coordination and location of assembly of extra-axonemal structures are less clear. We have discovered two cytoplasmic proteins in *Trypanosoma brucei* that are required for PFR formation, PFR assembly factors 1 and 2 (PFR-AF1 and PFR-AF2, respectively). Deletion of either PFR-AF1 or PFR-AF2 dramatically disrupted PFR formation and caused a reduction in the amount of major PFR proteins. The existence of cytoplasmic factors required for PFR formation aligns with the concept that processes facilitating axoneme assembly occur across multiple compartments, and this is likely a common theme for extra-axonemal structure assembly.

## Abstract (precis)

**Summary:** Two cytoplasmic proteins are shown to be required for paraflagellar rod formation, which are extra-axonemal structures found in the flagella of trypanosomes.

## INTRODUCTION

The eukaryotic flagellum is a well conserved organelle with multiple functions, which include providing a propulsive force and acting as a sensory platform (Moran et al., 2014). A flagellum consists of a microtubule axoneme surrounded by plasma membrane. The number and arrangement of microtubules in the axoneme can vary, with a motile flagellum typically containing nine outer microtubule doublets encircling a central pair of singlet microtubules to give a 9+2 arrangement, whereas a primary cilium (a term used interchangeably with flagellum) has a 9+0 axoneme, lacking the central pair. Flagellar proteomes of diverse organisms, and the recent genome-wide protein-localisation project (TrypTag) in *Trypanosoma brucei*, have shown that the flagellum is a complex organelle containing over 1000 proteins (Beneke et al., 2019; Broadhead et al., 2006; Dean et al., 2017; Hart et al., 2009; Narita et al., 2012; Ostrowski et al., 2002; Pazour et al., 2005).

Flagellum assembly is a complex, multi-site process involving three main compartments: (1) the cytoplasm, (2) the transition fibre-transition zone and (3) the flagellum. Protein components are synthesised in the cytoplasm, then sorted and directed to the flagellum via the transition fibre and transition zone, with many subsequently transported to the flagellum tip by the intraflagellar transport system before being incorporated into the flagellum structure (Langousis and Hill, 2014; Sánchez and Dynlacht, 2016). Not all flagellar proteins are transported into the flagellum as individual proteins. Dynein arms are large, highly structured complexes (consisting of 11–17 proteins) comprising heavy, intermediate and light chains that are attached to the A tubule of the outer microtubule doublet of the axoneme (Kobayashi and Takeda, 2012). Mutations in dynein proteins cause defective swimming in *Chlamydomonas* and primary ciliary dyskinesia in humans (Desai et al., 2018; Kobayashi and Takeda, 2012). In addition to the dynein proteins themselves, disruption of other proteins can cause the loss of the axonemal outer and inner dynein arms, resulting in flagellar motility defects. Investigation of these proteins, predominantly in *Chlamydomonas*, has led to the discovery of an ordered axonemal dynein assembly process that has three key steps each occurring in each of the three main compartments: (1) cytoplasmic assembly and maturation of the outer dynein arm complex, (2) transport of the complex into the flagellum and (3) docking of the complex to the microtubule doublet (Desai et al., 2018). Thus, although some components of the axoneme seem to travel to the flagellum tip as individual proteins, others are pre-assembled in the cytoplasm; however, all are influenced by the three main compartments required for flagellum assembly.

In many organisms there are additional extra-axonemal structures; for example, the outer dense fibres and fibrous sheath in mammalian sperm, mastigonemes in *Chlamydomonas*, vane structures found in protists such as the fornicate *Aduncisulcus paluster* and the paraflagellar rod (PFR) in *T. brucei* and other *Euglenozoa* (de Souza and Souto-Padrón, 1980; Hyams, 1982; Irons and Clermont, 1982a; Irons and Clermont, 1982b; Nakamura et al., 1996; Portman and Gull, 2010; Yubuki et al., 2016). When viewed using thin-section electron microscopy, outer dense fibres, the ventral vane of *A. paluster* and the PFR all have a striated appearance, suggesting a regular high-order structure (Farina et al., 1986; Portman and Gull, 2010; Woolley, 1971; Yubuki et al., 2016). The outer dense fibres of mammalian sperm are associated with the nine outer microtubule doublets in the principal piece of the sperm flagellum (Irons and Clermont, 1982a). Surrounding the axoneme and the outer dense fibres is the fibrous sheath, which is formed of two longitudinal columns that are attached to outer dense fibres 3 and 8 and are connected to each other by semi-circular transverse ribs (Eddy et al., 2003). The outer dense fibres contain at least 25 proteins, with a further nine known to localise to the fibrous sheath (Eddy et al., 2003; Petersen et al., 1999). The predominate proteins in the fibrous sheath are two A-kinase anchor family proteins, AKAP3 and AKAP4, which enable the fibrous sheath to act as a platform for signalling and metabolic pathways. Many proteins involved in glycolysis are associated with the fibrous sheath, for example isoforms of GAPDH and HK1 (Eddy et al., 2003). Disruption of the expression of outer dense fibre proteins, such as ODF2, and fibrous sheath proteins, including AKAP4, causes defects in outer dense fibre and fibrous sheath structure that impact sperm motility (Miki et al., 2002; Tarnasky et al., 2010; Zhao et al., 2018). These structures therefore likely provide mechanical support and also act as signalling and metabolic platforms, important for flagellar beat regulation.

Both the outer dense fibres and the fibrous sheath are assembled in the sperm flagellum once the axoneme has been built. The outer dense fibres are built in a proximal to distal direction along the flagellum, whereas the fibrous sheath is assembled in a distal to proximal direction, with the longitudinal columns assembled first before being connected by the transverse ribs (Irons and Clermont, 1982a,b). Little is known about the mechanism of the assembly of these structures; however, the deletion of ubiquitin-conjugating enzyme UBE2B results in sperm flagella that have a normal axoneme structure but disrupted positioning of the longitudinal columns (Escalier, 2003). During flagella regeneration in *Chlamydomonas*, mastigonemes appear on the new flagellum within 15 min of amputation of the old flagella, suggesting that these structures, as with the outer dense fibres and fibrous sheath, are assembled after the axoneme. Moreover, mastigonemes are not found at the base of newly assembled flagellum but instead are observed on the distal three-quarters of the regenerating flagellum (Nakamura et al., 1996).

In *Euglenozoa*, including *T. brucei*, the axoneme is accompanied by the extra-axonemal PFR. In *T. brucei* nearly 200 proteins have been found in the PFR, including the two most abundant components PFR1 and PFR2 (Dean et al., 2017; Portman et al., 2009). The PFR contains proteins such as adenylate kinases, cyclic nucleotide phosphodiesterases, and calmodulin indicating it has roles in both cAMP- and calcium-regulation that are likely to be relevant to flagellum beat regulation and to possible sensory functions. Hence, parallels can be drawn between the roles of the PFR and the fibrous sheath of sperm flagella (Ginger et al., 2013; Luginbuehl et al., 2010; Moran et al., 2014; Pullen et al., 2004).

Normally, the PFR lies parallel to the axoneme with the structure first appearing at a variable distance from the basal body, depending on the species, and then tapering towards the flagellum tip. The PFR has an intricate paracrystalline structure with three distinct domains (proximal, intermediate and distal) and is attached to the axoneme via microtubule doublets 4 and 7 (Farina et al., 1986; Portman and Gull, 2010). However, the PFR can vary dramatically in structure, with *Angomonas deanei* and *Strigomonas culicis* both having a very short and simplified PFR (Gadelha et al., 2005; Motta et al., 2013). The extra-axonemal PFR of *T. brucei* is assembled via PFR1 and PFR2 subunit incorporation at the distal tip of the growing flagellum, which lags behind axoneme assembly (Bastin et al., 1999b). The two most abundant proteins in the PFR, PFR1 and PFR2, are critical for its assembly. Loss of PFR1 and PFR2 components in *T. brucei*, or in the related kinetoplastid *Leishmania mexicana*, results in a loss of the PFR structure (Santrich et al., 1997; Bastin et al., 1998, 1999b; Maga et al., 1999) and a profound reduction in motility. To date only one other PFR protein, calmodulin, has been shown to have an important role in PFR assembly, with many minor protein components such as PFC3 and PAR1 not required for its assembly (Ginger et al., 2013; Lacomble et al., 2009).

The incorporation of axonemal proteins into the flagellum requires processes within three different compartments (cytoplasm, transition fibre-transition zone, flagellum). We know that extra-axonemal structure assembly occurs within the flagellum of cells such as trypanosomes and sperm (Bastin et al., 1999a; Irons and Clermont, 1982a,b). In trypanosomes, two non-PFR proteins have been identified to be important for PFR assembly, KIF9B and FOPL (Demonchy et al., 2009; Harmer et al., 2018). KIF9B is found in the basal body, pro-basal body, and axoneme, whereas FOPL is found at the transition fibres. RNAi-mediated knockdown of either of these two proteins causes severe defects in PFR assembly with flagellum sections showing either no PFR or accumulation of multiple PFR units (Demonchy et al., 2009; Harmer et al., 2018). The localisation of FOPL at the transition fibres shows that this compartment is also important for PFR formation. Given that PFR formation requires processes within the flagellum and at the transition fibres, are there processes in the cytoplasm that are required for extra-axonemal formation?

Here, we identified two cytoplasmic proteins, which together form a complex that is required for PFR formation. This suggests that assembly of extra-axonemal structures, as with the axoneme, requires processes in all three compartments. Presumably, such cytoplasmically localised phenomena reflect a need for the construction of major flagellar structures to be regulated and coordinated to ensure delivery of correct stoichiometric amounts of components during precise temporal windows and via particular transport systems.

## RESULTS

### Deletion of the *T. brucei* gene *Tb927.10.8870* causes slow growth and errors in cytokinesis

**Fig. 1.**
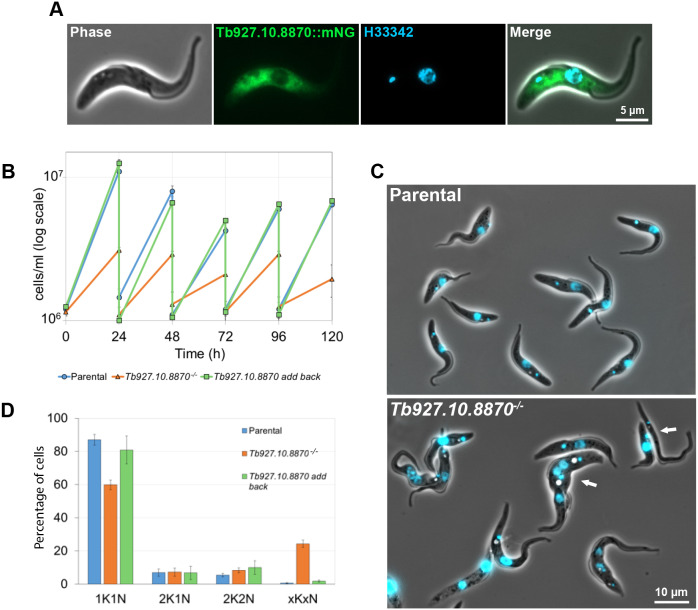
**Tb927.10.8870 is a cytoplasmic protein required for robust cell growth.** (A) Images of a live­ cell expressing Tb927.10.8870::mNG (green) stained with the DNA stain Hoechst 33342 (blue). Scale bar: 5 µm. (B) Growth curves of parental, *Tb927.10.8870^−/−^* and *Tb927.10.8870 add-back* cell lines over 120 h. Growth curves were performed in duplicate and the mean±s.d. is plotted. (C) Merge of phase-contrast and Hoechst-stain images of parental and *Tb927.10.8870^−/−^* live cells. White arrows indicate abnormal cell types. Scale bar: 10 µm. (D) Number of nuclei (N) and kinetoplasts (K) per cell was counted for 200 cells of parental, *Tb927.10.8870^−/−^* and *Tb927.10.8870 add-back* cell lines using fluorescence images of DNA stained with Hoechst 33342. All cells with abnormal numbers of K and N were counted as ‘xKxN’. Counts were performed in triplicate and the mean±s.d. is plotted.

We performed a deletion screen of potential actin interactors that included Tb927.10.8870, which is annotated in TriTrypDB as a myosin-like coiled-coil protein (Aslett et al., 2010). Tb927.10.8870 is an ∼34 kDa protein, which is predicted to be a coiled-coil protein that shares sequence identity with the taxilin domain model defined by Pfam (PF09728). Results from TrypTag, a global survey of trypanosome protein localisation, showed that the mNeonGreen (mNG)-tagged Tb927.10.8870 protein localises to the cytoplasm and is excluded from the flagellum and the nucleus (Dean et al., 2017). We confirmed this by endogenously tagging Tb927.10.8870 with mNG at its C-terminus and examining its localisation (Fig. 1A). Tb927.10.8870::mNG was restricted to the cytoplasm and had a patchy distribution, as seen previously, which was concentrated in the posterior half of the cell.

We investigated the function of Tb927.10.8870 by generating a cell line in which both alleles of the gene had been replaced with antibiotic resistance markers using a CRISPR/Cas9-based approach (Beneke et al., 2017). We did this in a newly developed cell line, which expresses the Cas9 nuclease, T7 RNA polymerase and Tet repressor from a single plasmid called pJ1339 and is therefore competent for both CRISPR/Cas9 genome editing and tetracycline-controlled inducible expression.

We were able to readily generate cell lines that were resistant to both selective drugs, and we confirmed the loss of both alleles of *Tb927.10.8870* and integration of the markers by PCR (Fig. S1A). However, we noticed that these null mutants took a longer time than normal to grow after the transfection, so we therefore measured the growth rate of the null mutant (Fig. 1B). Deletion of *Tb927.10.8870* caused cells to grow consistently slower than the parental cell line. Moreover, by light microscopy a number of abnormal forms were observed (Fig. 1C). The reduced growth rate and abnormal cell forms suggested that cytokinesis was potentially disrupted in this cell line. During the trypanosome cell cycle the kinetoplast (concatenated mitochondrial DNA) and the nucleus replicate and divide at specific time points, and their relative number in the cell indicates the cell cycle stage. To investigate if the cell cycle was disrupted we imaged the null mutant and parental cells with a fluorescent DNA stain and quantified the number of nuclei and kinetoplasts in each cell (Fig. 1D). There was a reduction in the percentage of cells with one kinetoplast and one nucleus in the null mutant, and an increase in the percentage of cells with an abnormal number of nuclei and kinetoplasts, indicating that there was a defect in cytokinesis in the null mutant.

To confirm that the changes we observed in the null mutant were due to the loss of Tb927.10.8870, we generated a Tb927.10.8870 add-back cell line by introducing a copy of the *Tb927.10.8870* gene into the null mutant with a 5′ Ty epitope tag (Bastin et al., 1996). We confirmed the expression and localisation of Tb927.10.8870 in the add-back cell by western blot and fluorescence microscopy (Fig. S1B,C). The Ty-tagged protein ran at the expected size on the western blot and its localisation was restricted to the cytoplasm, as observed with the mNG-tagged protein. The growth rate of the add-back cell line was similar to that of the parental cell line, and cells with abnormal numbers of nuclei and kinetoplasts were much less frequent than compared to the null mutant (Fig. 1D). This shows that the slow growth and cytokinesis defect of the null mutant were due to the loss of Tb927.10.8870 and unlikely to be due to an off-target effect.

### Tb927.10.8870 is required for PFR assembly

**Fig. 2.**
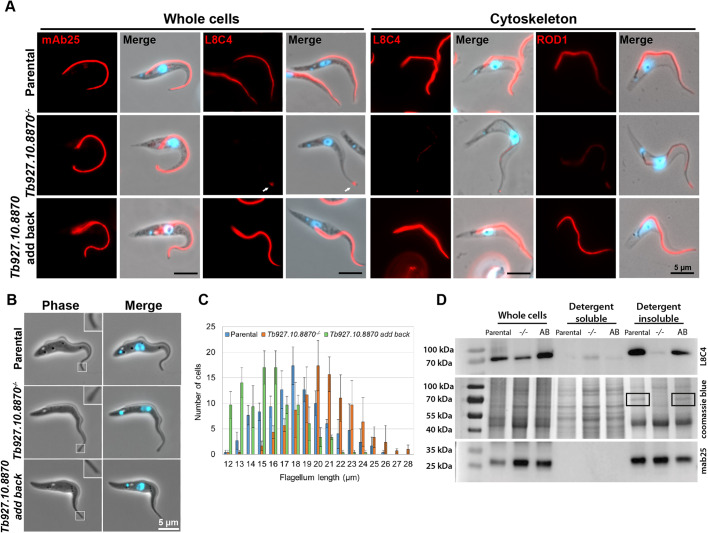
**PFR assembly is disrupted in *Tb927.10.8870^−/−^* mutant.** (A) Immunofluorescence was performed using the monoclonal antibodies mAb25 (recognising TbSAXO) and L8C4 (recognising PFR2) on methanol-fixed cells (whole cells), and L8C4 and ROD1 (recognising the distal-domain protein Tb5.20) on cytoskeleton preparations (cytoskeleton). Antibody labelling (red) and merge images (antibody, Hoechst 33342 and phase contrast) of parental, *Tb927.10.8870^−/−^* and *Tb927.10.8870 add-back* cell lines are shown. White arrow indicates bulge of PFR material at the flagellum tip. Scale bars: 5 µm. These are representative images from >100 cells/cytoskeletons observed for each labelling experiment. (B) Phase-contrast and merge (phase contrast and Hoechst 33342) images of parental, *Tb927.10.8870^−/−^* and *Tb927.10.8870 add-back* live cells, each with one nucleus and one kinetoplast. Insets show magnified views of the flagellar tip. Scale bar: 5 µm. (C) Histogram of flagellum length. Flagellum length was measured in 100 parental, *Tb927.10.8870^−/−^* and *Tb927.10.8870 add-back* 1% NP-40 extracted cells. Measurements were performed in triplicate and the mean ±s.d. is plotted. (D) Fractionations of parental, *Tb927.10.8870^−/−^* (−/−) and *Tb927.10.8870 add-back* (AB) cells using 1% NP-40. Western blotting was performed on detergent-soluble and detergent-insoluble fractions and whole cells using the monoclonal antibody L8C4, which recognises PFR2. The equivalent of 1×10^7^ cells was loaded per lane. mAb25 signal and the Coomassie Blue-stained gel were used as loading controls. The PFR bands in the Coomassie Blue-stained gel image are highlighted by black rectangles. Blots and gel image shown are representative of three experiments.

Careful examination of the light micrographs of null mutant cells showed that 52% (*n*=300) of cells with one flagellum had a bulge at the distal tip of the flagellum in comparison to only ∼3% of comparable parental (*n*=92) and add-back (*n*=165) cells (Fig. 2B). When null mutant cells were examined by scanning electron microscopy (SEM), the bulge at the tip of the flagellum was readily apparent (Fig. S2A). This suggests that flagellum assembly in these cells was disrupted. To determine whether there were changes in the trypanosome flagellum upon deletion of *Tb927.10.8870*, we stained the parental, null mutant and add-back cells with monoclonal antibodies that detect an axonemal component, TbSAXO (mAb25), and the PFR component PFR2 (L8C4) (Fig. 2A). In the parental, null mutant and add-back cells, mAb25 stained a linear structure in the flagellum that extended from close to the kinetoplast to the tip of the flagellum, correlating with the position of the flagellum axoneme. In the parental and add-back cells, L8C4 stained a linear structure within the flagellum from the point at which the flagellum exited the flagellar pocket and along the majority of the flagellum before fading towards the distal tip, which corresponds to the location of the PFR. However, in the null mutant the L8C4 signal in the flagellum was no longer evenly distributed, with patches of strong staining interspersed with regions of very weak staining, and often there was a strong signal at the distal tip of the flagellum that coincided with a bulge. This suggests that Tb927.10.8870 is important for PFR assembly but not axoneme assembly, and hence we named this protein PFR assembly factor 1 (PFR-AF1).

We further analysed the effect of PFR-AF1 deletion by generating detergent-resistant cytoskeletons of parental, null mutant and add-back cells, which were subsequently stained with L8C4 and ROD1, a monoclonal antibody against Tb5.20 (Woodward et al., 1994), a component of the distal domain of the PFR (Fig. 2A). In the null mutant cytoskeletons L8C4 signal was observed; however, the signal was much lower and had a patchy distribution in comparison to the L8C4 signal in parental and add-back cells. A similar pattern was observed with ROD1. The signal was weaker along the flagellum, with an accumulation of signal on the flagellum tip of null mutant cytoskeletons. Conversely, an even signal along the flagellum that faded towards the distal tip was seen in both the parental and add-back cytoskeletons. Together this provided further evidence that the loss of PFR-AF1 affected the stability of the PFR, with Tb5.20 and PFR2 signal intensity in the flagellum reduced on detergent treatment. We next measured the length of the flagellum in cells with one flagellum in the parental, null mutant and add-back cells (Fig. 2C). The parental cells had a mean flagellum length of 18.1 µm (*n*=100), whereas the null mutant had a slightly longer mean flagellum length, 19.9 µm (*n*=100) and the add-back cells had a shorter mean flagellum length, 16.4 µm (*n*=100). This suggests that there is a potential connection between PFR assembly and flagellum length.

Given the disruption to the PFR in the PFR-AF1 null mutants, we investigated the subcellular distribution of PFR2 by using the L8C4 monoclonal antibody on western blots of whole-cell, detergent-soluble, and detergent-insoluble lysates from the parental, null mutant and add-back cells (Fig. 2D). In the whole-cell lysates, the amount of PFR2 detected was lower in the null mutant than in the parental and add-back cells, confirming the immunofluorescence observation. In the insoluble fraction, there was little PFR2 detected in the null mutant, with the PFR band no longer visible in the Coomassie-stained gel. In addition, more PFR2 was detected in the soluble fraction of the null mutant than in the soluble fractions of parental and add-back cells (Fig. 2D, Fig. S2B). This again showed that the loss of PFR-AF1 disrupted PFR formation, with PFR2 not integrated into the PFR structure. The PFR2 detected in the detergent-soluble fraction ran at a consistently lower molecular weight than the PFR2 in the whole-cell lysate or detergent-insoluble fraction (Fig. 2D). This suggests that soluble PFR2 might be more susceptible to cleavage in the cell than PFR2 protein integrated into the PFR structure.

**Fig. 3.**
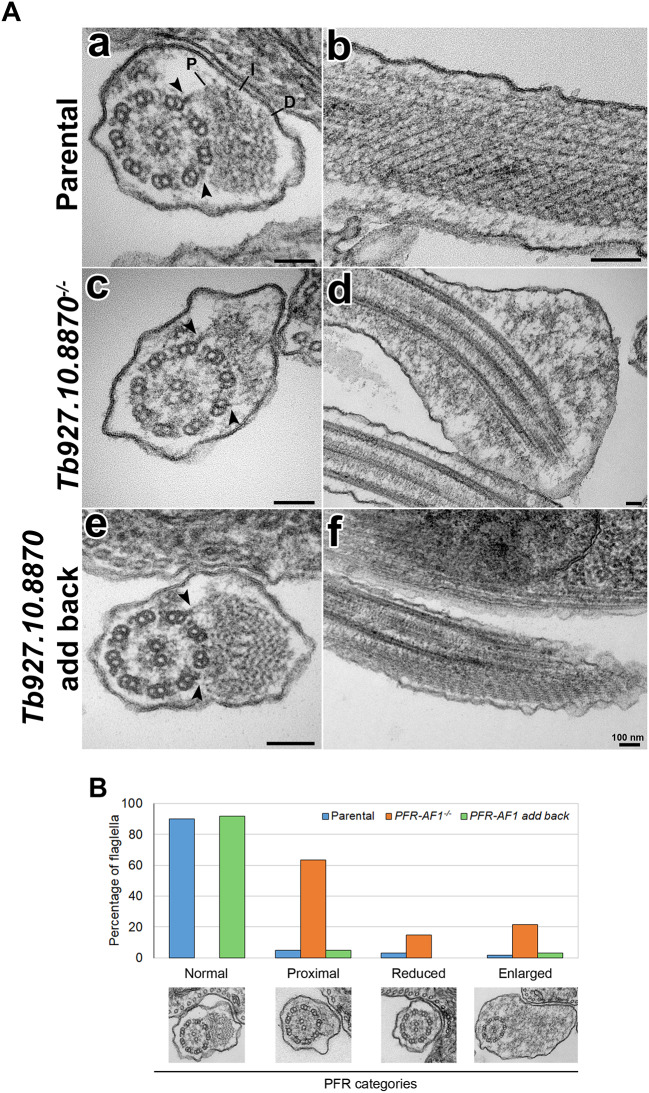
**PFR structure is perturbed in the *PFR-AF1^−/−^* mutant.** (A) TEM images of transversal (left) and longitudinal (right) flagellum sections of parental (a,b), *Tb927.10.8870^−/−^* (c,d) and *Tb927.10.8870 add-back* (e,f) cell lines. Arrows show the bridges that connect PFR to the axoneme. PFR domains are indicated in (a) as P (proximal), I (intermediate) and D (distal). Scale bars: 100 nm. (B) TEM observation of 60 random flagellum sections of parental, *PFR-AF1^−/−^* and *PFR-AF1 add-back* cell lines was used to separate PFR phenotypes into four categories: normal, proximal, reduced and enlarged. A representative TEM image is shown under the columns of each category.

To investigate the PFR assembly defect in more detail we examined the parental, null mutant and add-back cells by thin-section transmission electron microscopy (TEM) (Fig. 3A, a–f). In transverse sections across the flagellum of parental and add-back cells we observed the expected 9+2 microtubule axoneme with the PFR located alongside. The PFR was attached to microtubule doublets 4 and 7 by a linker structure, and the paracrystalline nature of the PFR was observed in both longitudinal and transverse sections. In the null mutant cells, the microtubule axoneme appeared intact and similar to that observed in the parental and add-back cells. In *T. brucei*, the central pair is aligned parallel to the PFR, and this alignment was unaffected in the null mutant (Gadelha et al., 2006). However, the PFR structure was severely affected in the null mutant (Fig. 3A, c–d). The bulge observed by light and fluorescence microscopy was clearly seen by TEM and consisted of a large amorphous collection of electron dense fibres that likely correspond to mis- or unassembled PFR components (Fig. 3A, d). However, in cross sections containing a small, misformed PFR the linkers connecting the PFR to the microtubule doublets 4 and 7 were still present, suggesting that PFR-AF1 was not required for linker assembly into the axoneme. To quantify the changes we observed in the PFR, we defined four categories of PFR structure: normal, proximal domain only, reduced and enlarged (Fig. 3B). For the parental and add-back cells the majority of flagellar cross sections had a normal PFR structure, whereas for the null mutant the majority of the cross sections had the proximal PFR domain only with a fraction also having a further reduced PFR or an enlarged PFR. This provides further evidence that PFR-AF1 was required for the correct formation of the PFR.

### PFR-AF1 interacts with a cytoplasmic coiled-coil protein

**Fig. 4.**
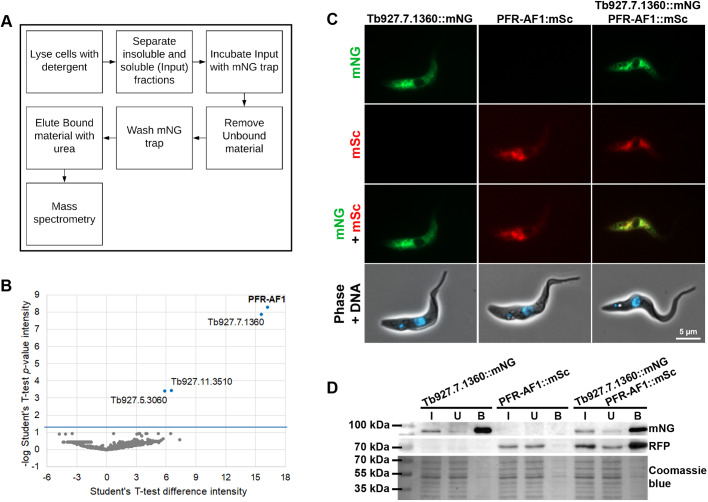
**Tb927.7.1360 is a binding partner of PFR-AF1.** (A) Schematic of experimental procedure for immunoprecipitation and mass spectrometry from cells expressing either PFR-AF1::mNG or soluble mNG. (B) Identification of PFR-AF1-interacting proteins by mass spectrometry. Volcano plot of Student's *t*-test difference in intensity versus *P*-value of intensity for the PFR-AF1::mNG versus soluble mNG input. Graph represents the combined analysis of three independent experiments. (C) Fluorescence images of cell lines expressing either Tb927.7.1360::mNG (green), PFR-AF1::mSc (red) or both. Scale bar: 5 µm. (D) Western blots of immunoprecipitations using the mNG trap with cell lines expressing Tb927.7.1360::mNG, PFR-AF1::mSc and both Tb927.7.1360::mNG and PFR-AF1::mSc. Western blotting was performed on the input (I), unbound (U), and bound (B) material for each cell line using mNG to detect Tb927.7.1360::mNG and anti-RFP to detect PFR-AF1::mSc, with a Coomassie Blue-stained gel to show total protein present. The input is the cell lysate applied to the mNG trap. The unbound material is all the proteins that did not bind to the mNG trap and were washed off. The bound material is the proteins that bound to the mNG trap and were not washed off. For the bound material, 10% of the total input was loaded. PFR-AF1::mSc was only detected when Tb927.7.1360::mNG was present. Blots and gel image shown are representative of three experiments.

The cytoplasmic localisation of PFR-AF1 suggested that this protein is unlikely to be involved in the transport of PFR protein components into and along the flagellum or their incorporation into the flagellum. The assembly of certain axonemal components, such as the outer dynein arms, occurs in the cytoplasm before the assembled complex is transported into the flagellum (Desai et al., 2018). To investigate whether PFR-AF1 interacts with any PFR proteins we performed immunoprecipitation assays on cells expressing either PFR-AF1::mNG or soluble mNG. Whole-cell lysates were prepared from these cells and the mNG proteins were captured using the mNG-trap method, followed by mass spectrometry (Fig. 4A). We performed these immunoprecipitations in triplicate and compared the enrichment of proteins precipitating with PFR-AF1::mNG versus those precipitating with soluble mNG only (Fig. 4B). Four proteins were significantly enriched in PFR-AF1::mNG samples in comparison to the soluble mNG samples. As expected PFR-AF1 was the most significantly enriched protein, with Tb927.7.1360 also enriched to a similar degree (Fig. 4B). Two further proteins, Tb927.5.3060 and Tb927.11.3510, were enriched but not to the same degree as Tb927.7.1360 and had low Mascot scores (<12), suggesting low confidence of identification in the mass spectrometry analysis. However, our approach did not identify any known PFR proteins.

An alternative explanation for the effect of PFR-AF1 deletion on PFR assembly is that PFR-AF1 is a cytoplasmic factor required for the specific translation of PFR proteins and its loss results in reduced PFR protein expression. To test this hypothesis we treated cells with MG132, an inhibitor of the proteasome, in order to stabilise proteins that would normally be degraded without having an effect on translation. If PFR-AF1 is important for PFR protein translation, then the addition of MG132 to the null mutant would not alter the amount of PFR protein in the cell. However, after addition of MG132 to the null mutant for 8 h we saw an increase in the amount of PFR2 relative to mAb25 signal, although the amount of PFR2 did not reach parental levels (Fig. S2C,D). This suggests that PFR2 was being degraded in the PFR-AF1 null mutant in part by the proteasome, and that PFR-AF1 was important for PFR protein stability.

### Tb927.7.1360 is also required for PFR assembly

Given that Tb927.7.1360 was found to be significantly enriched in the PFR-AF1 immunoprecipitation relative to immunoprecipitation with soluble mNG and that its localisation, as reported in the TrypTag database, is similar to that of PFR-AF1, we decided to focus on this protein. Tb927.7.1360 is predicted to form a single coiled-coil domain and have a localisation pattern restricted to the kinetoplastids, and has no known homologues in other organisms (https://tritrypdb.org; Aslett et al., 2010). Our immunoprecipitation assays to find proteins binding PFR-AF1::mNG showed that Tb927.7.1360 interacts with PFR-AF1. Next, we wanted to confirm this interaction was reciprocal by determining whether PFR-AF1 would be co-immunoprecipitated with Tb927.7.1360. We generated three cell lines, one expressing Tb927.7.1360 endogenously tagged with mNG, another expressing PFR-AF1 tagged with mScarlet (mSc) and one expressing both Tb927.7.1360::mNG and PFR-AF1::mSc. Both the tagged proteins had the expected cytoplasmic localisation (Fig. 4C). Cell lysates from these three cell lines were used for immunoprecipitation using the mNG-trap method, with the tagged proteins detected by western blotting using mNG for Tb927.7.1360::mNG and anti-RFP for PFR-AF1::mSc. For the cell line expressing Tb927.7.1360::mNG only, this protein was found in the input (I) and mNG-trap bound (B) fractions, showing that the mNG-tagged protein was efficiently bound by the mNG trap. For the cell line expressing PFR-AF1::mSc only, this protein was found in the input (I) and unbound (U) fractions with no enrichment in the bound (B) fraction, showing that PFR-AF1::mSc did not interact with the mNG trap. For the cell line expressing both Tb927.7.1360::mNG and PFR1-AF1::mSc both proteins were present in the bound (B) fraction, indicating that these two proteins specifically interact (Fig. 4D).

**Fig. 5.**
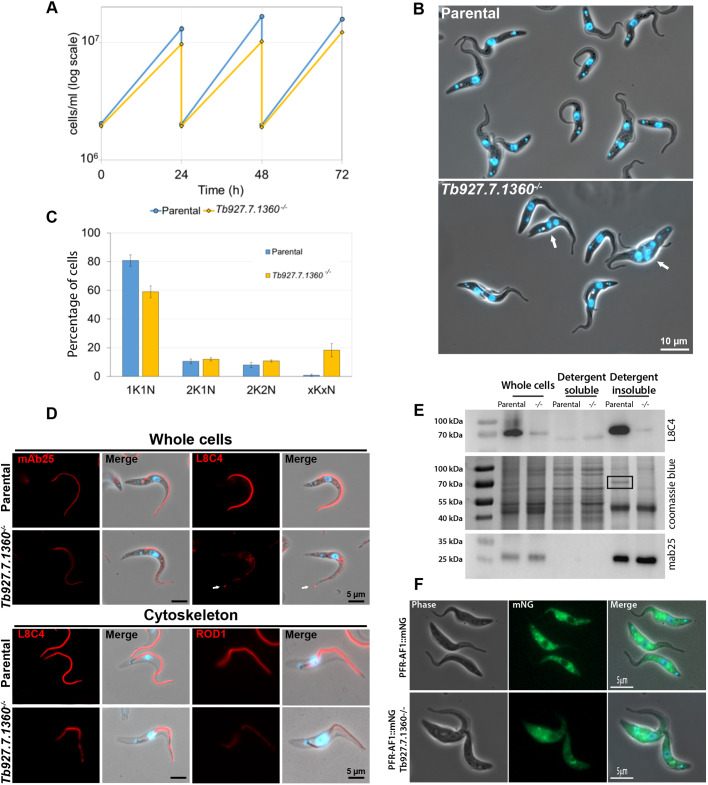
**Tb927.7.1360 deletion disrupts PFR formation.** (A) Growth curves of parental and *Tb927.7.1360^−/−^* cell lines over 72 h. Growth curves were performed in duplicate and mean±s.d. is plotted. (B) Merge of phase-contrast and Hoechst images of parental and *Tb927.7.1360^−/−^* live cells. White arrows indicate abnormal cells. Scale bar: 10 µm. (C) Number of nuclei (N) and kinetoplasts (K) were counted in 200 cells of parental and *Tb927.7.1360^−/−^* cell lines using fluorescence images of DNA stained with Hoechst 33342. All cells with abnormal numbers of K and N were counted as ‘xKxN’. Counts were performed in triplicate and mean ±s.d. is plotted. (D) Immunofluorescence was performed using the monoclonal antibodies mAb25 (recognising TbSAXO) and L8C4 (recognising PFR2) on methanol-fixed cells (whole cells) and L8C4 and ROD1 (recognising the distal domain protein Tb5.20) on cytoskeleton preparations. Antibody labelling (red) and merge images (antibody, Hoechst 33342 and phase contrast) of parental and *Tb927.7.1360^−/−^* cell lines are shown. These are representative images from >100 cells/cytoskeletons observed for each labelling experiment. Scale bars: 5 µm. (E) Fractionations of parental and *Tb927.7.1360^−/−^* (−/−) cells using 1% NP-40. Western blotting was performed on detergent-soluble and detergent-insoluble fractions and whole cells using the monoclonal antibody L8C4, which recognises PFR2. The equivalent of 1×10^7^ cells was loaded per lane. mAb25 signal and the Coomassie Blue-stained gel were used as loading controls. The PFR band in the Coomassie Blue-stained gel image is highlighted by a black rectangle. Blots and gel image shown are representative of three experiments. (F) Images of live parental or Tb927.7.1360^−/−^ cells expressing PFR-AF1::mNG (green) with the DNA stained with Hoechst 33342 (blue). Scale bars: 5 μm.

To understand the function of Tb927.7.1360, we generated a cell line in which both alleles of *Tb927.7.1360* were replaced with antibiotic resistance markers. Loss of the *Tb927.7.1360* open reading frame and integration of the resistance makers was confirmed by PCR (Fig. S3A). The growth rate of the Tb927.7.1360 null mutant was compared to that of the parental cell line and we found that it was consistently slower growing (Fig. 5A). Imaging of the Tb927.7.1360 null mutant stained with the DNA stain Hoechst 33342 showed a similar range of cell cycle defects as those observed following deletion of PFR-AF1 (Fig. 5B,C). In addition, we noticed that there was a bulge at the tip of the flagellum of ∼52% (*n*=240) of Tb927.7.1360 null mutant cells with one flagellum, as also previously seen in cells without PFR-AF1. Taken together, this suggested that Tb927.7.1360 was likely affecting PFR formation.

We investigated the effect of Tb927.7.1360 deletion on the structure of the axoneme and PFR by staining the cells with the axoneme-binding antibody mAb25 and the PFR-binding antibody L8C4 (Fig. 5D). There was no difference in staining pattern of mAb25 between the parental and Tb927.7.1360 null mutant cells, as was also the case for PFR-AF1 null mutant cells. In the parental cells L8C4 strongly stained the PFR within the flagellum. However, in the Tb927.7.1360 null mutant the L8C4 signal in the flagellum was no longer evenly distributed with patches of stronger staining interspersed with regions of very weak staining. We investigated the defect in PFR formation further by staining cytoskeletons with L8C4 and ROD1 antibodies (Fig. 5D). In parental cytoskeletons L8C4 gave a strong signal along the flagellum, whereas in the Tb927.7.1360 null mutant the signal was weaker with distinct gaps. The ROD1 signal was distributed along the flagellum, fading towards the distal tip in parental cytoskeletons; however, in the Tb927.7.1360 null mutant cytoskeletons the ROD1 signal was weaker, with a less even distribution. Given the effect Tb927.7.1360 had on PFR assembly, we named this protein PFR assembly factor 2 (PFR-AF2). To complement the microscopy, we used detergent to fractionate cells into soluble and insoluble fractions, which were analysed by western blotting with L8C4 (Fig. 5E). Deletion of PFR-AF2 reduced the amount of PFR2 in the cells, with a marked reduction in PFR2 seen in the insoluble fraction, and the loss of the PFR band in the Coomassie-stained gel. The similar phenotypes observed on deletion of PFR-AF1 and PFR-AF2, and the interaction between the two proteins, suggest that these proteins work together to enable PFR formation. To investigate the interaction of PFR-AF1 and PFR-AF2 further, we deleted both alleles of the gene encoding PFR-AF2 in a cell line expressing PFR-AF1::mNG (Fig. S3B, Fig. 5F). In cells expressing PFR-AF1::mNG (Fig. 1A, 4C) and the cells expressing PFR-AF2::mNG (Fig. 4C), PFR-AF1 localisation was restricted to the cytoplasm and excluded from the nucleus. However, the localisation of PF-AF1::mNG was altered in the cells in which PFR-AF2 was deleted, with PFR1-AF::mNG now found in both the cytoplasm and the nucleoplasm, but not the nucleolus (Fig. 5F). The change in localisation of PFR-AF1 on PFR-AF2 deletion provides further evidence that these proteins interact.

## DISCUSSION

Extra-axonemal structures are found in many eukaryotic flagella including the outer dense fibres and fibrous sheath of sperm and the mastigonemes of *Chlamydomonas*. Initial research on these structures described their structure and assembly kinetics, and was followed by later work cataloguing and functionally analysing their constituent parts (Eddy et al., 2003; Irons and Clermont, 1982a,b; Nakamura et al., 1996; Ringo, 1967; Witman et al., 1972); however, little work has focussed on the mechanism of assembly of these structures. Due to the lack of ribosomes in the flagellum, all the proteins required for flagellar assembly must undergo a multi-step process before being finally incorporated into the flagellum, including translation in the cytoplasm, assembly and post-translational modification if required, sorting and delivery to the flagellum, transport along the flagellum, and assembly into the flagellum structure. These processes occur across three main compartments, the cytoplasm, the transition fibre-transition zone, and the flagellum. The assembly of extra-axonemal structures including the PFR and the outer dense fibres is likely to follow a similar pattern (Bastin et al., 1999b).

Here, we have shown that two proteins, PFR-AF1 and PFR-AF2, form a cytoplasmic complex specifically important for PFR assembly. These are the first cytoplasmic proteins to be associated with PFR formation. Deletion of either of these proteins resulted in similar phenotypes; the null mutants had a reduced amount of PFR2, were unable to assemble an intact PFR, grew more slowly, and there was a higher proportion of abnormal cells observed in the null mutant population. To date the only other proteins important for PFR formation are components of either the PFR itself (PFR1, PFR2, calmodulin), the axoneme and basal body (KIF9B), or the transition fibres (FOPL) (Bastin et al., 1998, 1999a; Demonchy et al., 2009; Ginger et al., 2013; Harmer et al., 2018; Maga et al., 1999; Santrich et al., 1997). The deletion of both PFR-AF1 and PFR-AF2 resulted in the accumulation of PFR proteins at the tip of the flagellum, showing that PFR proteins were still able to be trafficked into the flagellum and transported to the flagellum tip but, once there, they were not effectively assembled into the PFR structure. This suggests either that the action of PFR-AF1 and PFR-AF2 is required for PFR proteins to be competent for PFR formation, or that without PFR-AF1 or PFR-AF2 not all PFR components were able to reach the flagellum, disrupting PFR formation.

The residual PFR structure observed by TEM when PFR-AF1 was deleted was similar to that observed with PFR2 RNAi knockdown. However, PFR2 was still present in the PFR of the flagellum in the null mutants, so it is likely that composition of this residual structure is different to that observed in the PFR2 RNAi mutant (Bastin et al., 1998). Interestingly, in TEM cross sections of the flagellum in the KIF9B, calmodulin and FOPL RNAi mutants there are often multiple, individually discernible PFR cross sections that, though disordered, are similar in outline and size to a normal PFR cross section, suggesting that the ability to assemble a PFR-type structure is present and that the failure results from a lack of integration of these structures into a single continuous PFR alongside the axoneme (Demonchy et al., 2009; Ginger et al., 2013; Harmer et al., 2018). However, in the PFR-AF1 and PFR-AF2 deletion mutants a normal sized PFR structure was never observed, suggesting that the cytoplasmic PFR-AF1 and PFR-AF2 act upstream of KIF9B, calmodulin and FOPL in the PFR formation process. This fits well with the concept of flagellum assembly being composed of multiple processes across different locations, and suggests that FOPL, which localises to transitional fibres on the basal body, may act to regulate entry of PFR proteins into the flagellum with KIF9B and calmodulin required for their assembly within the flagellum.

In sperm, the ubiquitin conjugating enzyme UBE2B was shown to be important for the positioning and assembly of the longitudinal columns of the fibrous sheath (Escalier, 2003). Addition of ubiquitin can have a range of effects on a protein, including targeting it for degradation or altering its location in the cell; however, the specific function of ubiquitylation in fibrous sheath assembly is unknown. The TrypTag project has identified two ubiquitin-associated proteins that localise to the axoneme, and others that localise to the flagellar cytoplasm, but none specific for the PFR (Dean et al., 2017). Additionally, no further proteins have been identified to date that are important for assembly of the sperm extra-axonemal structures beyond the known integral components of those structures. However, interrogating the function of proteins involved in outer dense fibre and fibrous sheath assembly in sperm is difficult because these processes take place in terminally differentiated cells. Therefore, the PFR and its assembly mechanism provides a paradigm that will be useful when considering extra-axonemal structure assembly in other organisms.

The PFR has a regular, ordered structure and it is unlikely that this structure would arise spontaneously, suggesting that additional proteins are required to assist its assembly. Based on the assembly model outlined above, one can conjecture a number of different functions for such additional proteins including cytoplasmic assembly, post-translational modification, transport, and flagellar-based chaperones. As the PFR contains nearly 200 proteins (Dean et al., 2017), it is unlikely that each would have its own set of specific associated factors; therefore, it is more likely that PFR-AF1 and PFR-AF2 would be employed in assisting the assembly, modification or transport of multi-subunit complexes that are then incorporated into the PFR. This has clear parallels with dynein arm assembly. The assembly mechanism for the dynein arms that drive and coordinate flagellum movement has been well studied. This process has a cytoplasmic step during which the dynein arm proteins are folded and assembled into a complex before undergoing further maturation and delivery to the flagellum (Desai et al., 2018). This cytoplasmic step requires an expanding list of dynein-arm assembly factors [currently 10 are known: DNAAF1, DNAAF2, DNAAF3, MOT48, HEATR2, LRRC6, DNAAF4, PIH1D3/Twister, SPAG1 and ZMYND10 (Cho et al., 2018; Desai et al., 2018; Horani et al., 2012; Loges et al., 2009; Mitchison et al., 2012; Olcese et al., 2017; Omran et al., 2008; Tarkar et al., 2013)] that act as chaperones to ensure successful folding and assembly of the dynein arm complex. In the PFR-AF1 null mutant, PFR1 and PFR2 protein amount increased when the cells were treated with the proteasome inhibitor MG132 suggesting that, without PFR-AF1, PFR1 and PFR2 were unstable and degraded. This phenomenon is also observed for dynein heavy chains when DNAAF proteins, such as DNAAF3, are depleted (Mitchison et al., 2012).

The role of the PFR-AF1–PFR-AF2 complex in PFR formation still needs to be defined. The cytoplasmic localisation of these proteins and the possible roles for PFR-AFs discussed above may indicate that PFR-AF1 and PFR-AF2 are involved in the assembly of multi-subunit complexes. For example, do PFR-AF1 and PFR-AF2 act as a scaffold onto which PFR proteins can assemble before disengaging to allow the PFR unit to be transported to the flagellum? An enrichment of PFR proteins was not seen in the PFR-AF1 immunoprecipitation suggesting that any interaction between the PFR-AF1–PFR-AF2 complex and PFR proteins is likely to be transient and that the methods used here were not sensitive enough to detect such an interaction. Moreover, we do not know which of the nearly 200 PFR proteins are present in these putative PFR protein complexes. Although there is evidence for interaction between PFR proteins, none of these interactions involve PFR1 and PFR2 (Lacomble et al., 2009; Portman and Gull, 2010). There is also the potential for multiple types of assembly unit as the composition of the proximal domain and mid/distal domain are different, with the former able to form without PFR2.

PFR-AF1 and PFR-AF2, which have respective molecular masses of 34 kDa and 42 kDa, are predicted to form coiled coils. PFR-AF2 is restricted to the kinetoplastids, whereas PFR-AF1 was identified as containing a taxilin domain, a domain implicated in vesicular trafficking by binding to syntaxins. In trypanosomes, many proteins involved in vesicular trafficking are enriched on structures between the nucleus and flagellar pocket. As such, the cytoplasmic localisation of PFR-AF1 is not consistent with a role in vesicular trafficking, and this domain may therefore have evolved to bind a different set of partners in the kinetoplastids. However, a cytoplasmic localisation does not preclude a role in vesicular trafficking, and further work is required to clarify the function of PFR-AF1 and PFR-AF2. The PFR is specific to *Euglenozoa* but it can vary in appearance greatly between different species, with *A.* *deanei* and *S.* *culicis* having a much-reduced PFR structure that does not contain PFR2 (Gadelha et al., 2005; Motta et al., 2013). However, both PFR-AF1 and PFR-AF2 are conserved in these species and it is likely that even with a reduced PFR these proteins are required for its correct formation.

In summary, our data fit with the concept of multiple flagellum assembly processes occurring across different cellular compartments. The discovery of a set of positioned processes for extra-axonemal structures in *T. brucei* that is analogous to processes in the assembly of other eukaryotic flagella suggests that this is a common mechanism to deal with complications of building a complex structure whose site of assembly can be many microns from the cytoplasm.

## MATERIALS AND METHODS

### Cell culture

*Trypanosoma brucei* TREU927 procyclic forms containing the plasmid pJ1339 that expresses T7 RNA polymerase, Tet repressor and Cas9 were grown at 28°C in SDM-79 medium supplemented with 10% FCS (Brun and Schönenberger, 1979). Cell concentration was determined in a Z2 Coulter Counter particle counter.

### Generation of deletion constructs, tagging constructs and add-back constructs

Constructs for endogenous gene tagging and gene deletion mediated by CRISPR-Cas9 were generated as described by Beneke et al. (2017). To generate the *Tb927.10.8870* add-back construct, the *Tb927.10.8870* open reading frame was cloned into plasmid pJ1313 using the SpeI and BamHI restriction sites, which resulted in a fusion protein with an N-terminal Ty tag. pJ1313 is a modified version of p3605 (de Freitas Nascimento et al., 2018) in which additional SpeI sites have been removed and the 3Ty::GS::mNG::GS::3Ty cassette has been cloned into the HindIII/BamHI sites. This plasmid supports constitutive gene expression and enables the generation of proteins tagged with 3Ty and/or mNG at either the N- or C-terminus. All constructs were electroporated using Nucleofector 2b device and Program X-001 (Dean et al., 2015).

### Electron microscopy

#### TEM

Cells were fixed by addition of glutaraldehyde into the cultures to a final concentration of 2.5%. Cells were harvested by centrifugation at 800 ***g*** for 5 min and primary fixative (2.5% glutaraldehyde, 2% formaldehyde and 0.1 M sodium phosphate buffer, pH 7.0) was added without disturbing the pellet. After 1 h at room temperature, samples were washed three times in 0.1 M sodium phosphate buffer, pH 7, for 5 min and post-fixed in 1% osmium tetroxide in 0.1 M sodium phosphate buffer, pH 7, for 90 min at room temperature under agitation. Samples were washed in distilled water three times for 5 min and stained in 2% uranyl acetate for 12 h at 4°C. Samples were washed three times in water and dehydrated in increasing concentrations of acetone (30%, 50%, 70%, 90% v/v in distilled water, followed by three times in 100% acetone) and embedded in Agar-100 resin (Agar Scientific). Thin sections were post-stained in 2% uranyl acetate and 3% lead citrate. Images were obtained using a Hitachi H-7650, FEI Tecnai G2 Spirit or Jeol 1400Flash, operated at 120 kV.

#### SEM

Fixation was performed by adding glutaraldehyde into the cell culture to final concentration of 2.5%. Cells were harvested by centrifugation at 800 ***g*** for 5 min and primary fixative was added (2.5% glutaraldehyde in PBS). After 2 h, cells were washed twice in PBS and settled onto round glass coverslips for 5 min. Coverslips were washed two times in PBS and sample dehydration was performed using increasing concentrations of ethanol (30%, 50%, 70% and 90% v/v in distilled water, followed by three times in 100% ethanol) for 5 min in each step. Samples were dried using a critical point dryer. Coverslips were mounted onto SEM stubs using silver DAG (Agar Scientific) and coated with gold using a sputter coater. Images were acquired on a Hitachi S-3400N scanning electron microscope.

#### Antibodies

The primary antibodies used in this work and their dilutions were as follows: monoclonal mNeonGreen antibody (32F6; Chromotek) diluted 1:100 for western blotting, monoclonal RFP antibody (6G6; Chromotek) diluted 1:1000 for western blotting, monoclonal BB2 (Bastin et al., 1996) diluted 1:1000 for western blotting or 1:100 for immunofluorescence, monoclonal L8C4 (Kohl et al., 1999) diluted 1:1000 for western blotting or 1:200 for immunofluorescence, monoclonal mAb25 (Dacheux et al., 2012; provided by Prof. Derrick Robinson, University of Bordeaux, France) diluted 1:100 for immunofluorescence and monoclonal ROD1 (Woods et al., 1989; the Rod1 hybridoma cell line was a kind gift from Prof. Keith Gull, University of Oxford, UK) diluted 1:200 for immunofluorescence. Secondary antibodies used in this work and their dilutions were as follows: anti-mouse IgG conjugated to Alexa Fluor 546 (A11030; Invitrogen) diluted 1:200 for immunofluorescence, anti-mouse IgM conjugated to Alexa Fluor 546 (A21045; Invitrogen) diluted 1:250 for immunofluorescence and anti-mouse IgG conjugated to peroxidase (715-035-150; Jackson ImmunoResearch) diluted 1:1000 for western blotting.

#### Cell fractionation

2×10^6^ cells were harvested by centrifugation at 800 ***g*** for 5 min, washed in PBS and incubated with 50 µl of 1% NP-40 in PEME (0.1 M PIPES pH 6.9, 2 mM EGTA, 1 mM MgSO_4_ and 0.1 mM EDTA) containing protease inhibitors (Roche) for 5 min on ice. To obtain whole-cell samples after the treatment, 50 µl of 2× Laemmli buffer was added to the cell lysate. Soluble and insoluble fractions were separated by centrifugation at 20,000 ***g*** for 10 min at 4°C. To the soluble fraction, 50 µl of 2× Laemmli buffer was added. The insoluble fraction was resuspended in 50 µl of 1% NP-40 in PEME and 50 µl of 2× Laemmli buffer. All samples were incubated at 100°C for 5 min. For proteaseome inhibition experiments, MG132 (Sigma-Aldrich) was added to cells at a concentration of 20 µM for 8 h before preparation of whole-cell samples.

#### Western blotting

1×10^6^ or 2×10^7^ cell equivalents were loaded onto a 12% SDS–PAGE gel. After transfer, membranes were blocked with blocking solution (3% milk and 0.05% Tween 20 in PBS) for 1 h, incubated with primary antibody diluted in blocking solution for 1 h, washed three times in blocking solution and incubated with secondary antibody diluted in blocking solution for 1 h. Proteins were detected by ECL. Protein band signal intensity was measured in ImageJ (NIH, MD), the background was subtracted and then the signal normalised to the mAb25 loading control.

#### Immunofluorescence microscopy

Cells were harvested by centrifugation at 800 ***g*** for 5 min, washed in PBS and settled onto glass slides for 5 min. For whole-cell preparations, cells were fixed in −20°C methanol for 20 min and rehydrated in PBS for 10 min. For cytoskeleton preparations, cells were first treated with 1% NP-40 in PEME for 5 min, washed in PBS and fixed in −20°C methanol for 20 min. After fixation, the slides were blocked for 1 h at room temperature with blocking solution (1% BSA in PBS) and incubated in primary antibody diluted in blocking solution for 1 h. After washing three times in PBS, slides were incubated in secondary antibody diluted in blocking solution for 1 h. After three washes in PBS, slides were incubated in 20 μg/ml Hoechst 33342 (Sigma-Aldrich) in PBS, washed in PBS and mounted before imaging. For live-cell microscopy, cells were washed three times in PBS supplemented with 10 mM glucose and 46 mM sucrose (vPBS). In the second wash, DNA was stained using 10 µg/ml Hoechst 33342. After the third wash, cells were re-suspended in vPBS, settled onto glass slides, mounted with a coverslip and imaged immediately. Images were taken using a Zeiss Axio Imager.Z1 microscope equipped with an ORCA-Flash 4.0 CMOS camera using a Plan-Apochromat 100×/1.4 NA oil objective. Images were acquired and analysed with ZEN 2 PRO software and assembled for publication in Adobe Photoshop CS6.

#### Immunoprecipitation

2×10^8^ cells were harvested by centrifugation at 800 ***g*** for 5 min, washed in PBS and resuspended into 1 ml of lysis buffer (0.2% NP-40, 10 mM Tris–HCl pH 7.5 and 150 mM NaCl) for 5 min on ice. Soluble proteins were separated by centrifugation at 16,000 ***g*** for 10 min at 4°C. 25 µl of mNeonGreen-trap magnetic agarose previously washed in washing buffer (10 mM Tris–HCl pH 7.5 and 150 mM NaCl) were incubated with 500 µl soluble protein lysate (final concentration of 10^8^ cell equivalents/ml) for 30 min at 4°C tumbling end-over-end. Beads were magnetically separated and washed twice with washing buffer. Samples were eluted into 100 µl 1× Laemmli buffer for western blotting analysis or into 50 µl of 4 M urea for mass spectrometry analysis.

#### Mass spectrometry

Eluates from the immunoprecipitation were reduced, alkylated and then digested overnight with trypsin. The peptides were separated on an Ultimate 3000 UHPLC system and then directly electrosprayed into the coupled QExactive mass spectrometer. The raw data was acquired on the mass spectrometer in a data-dependent mode and full-scan MS spectra were acquired in the Orbitrap. The raw data was analysed using MaxQuant (version 1.5.3.8) with the integrated Andromeda search engine. For protein identification, peak lists were searched against a *T. brucei* database and a list of common contaminants. Protein and PSM false discovery rates were set at 0.01. The protein group output file was them imported to Perseus 1.5.2.4, to perform a Student’s *t*-test.

## Supplementary Material

Supplementary information
