# Supplementary material for: Control of assembly of extra-axonemal structures: the paraflagellar rod of trypanosomes
Source: J Cell Sci. 2020 May 27;133(10):jcs242271. doi: 10.1242/jcs.242271 (PMC7272336; doi:10.1242/jcs.242271)
Supplement: Supplementary information [file joces-133-242271-s1.pdf]

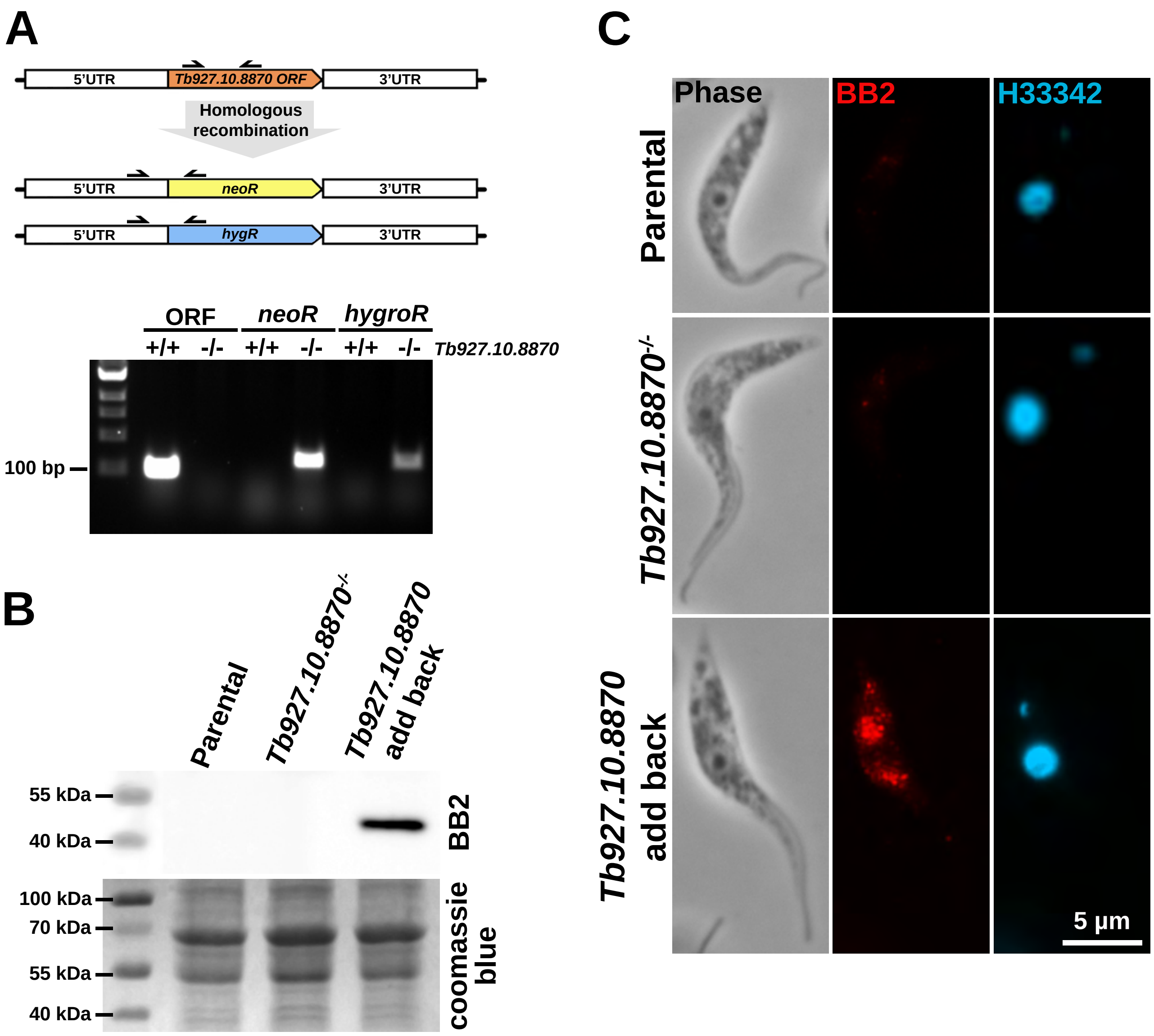

Figure S1. (A) Schematic and PCR confirmation of Tb927.10.8870 gene deletion. gDNA from Tb927.10.8870-/- mutant and the parental cells was analysed by PCR. PCR confirmed that Tb927.10.8870 ORF was no longer present in the null mutant and that the resistance markers had integrated correctly. (B) Confirmation of Ty::Tb927.10.8870 expression in add back cell line. Western blot was performed on whole cell lysates of parental, Tb927.10.8870-/- and Tb927.10.8870 add back cell lines using monoclonal antibody BB2 that recognises the Ty tag. Coomassie stained gel was used as a loading control. (C) Confirmation of Ty::Tb927.10.8870 localisation to the cytoplasm in the add back cell line. Immunofluorescence was performed using the BB2 antibody on methanol fixed whole cells. Phase, BB2 labelling (red) and merge images (antibody, Hoechst 33342 and phase) of parental and Tb927.10.8870-/- and Tb927.10.8870 add back cell lines are shown.

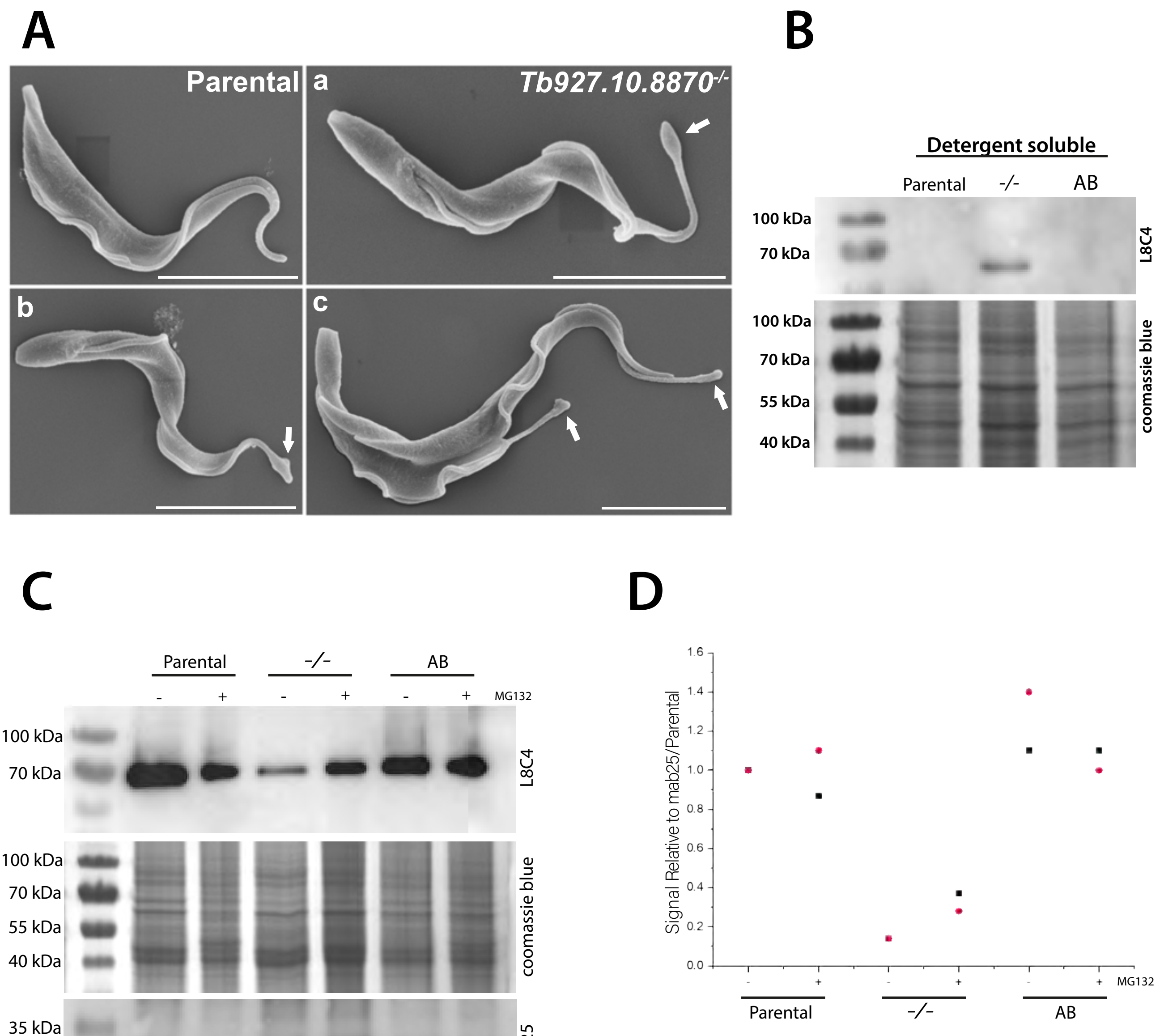

	Parental (-)	Parental (+MG132)	-/- (-)	-/- (+MG132)	AB (-)	AB (+MG132)
■	1	0.87	0.14	0.37	1.1	1.1
●	1	1.1	0.14	0.28	1.4	1

Figure S2. (A) SEM images of parental and Tb927.10.8870-/- cells. The bulge is clearly present at the tip of the flagellum White arrows indicate the bulge at the tip of the flagellum. (B) Western blot of detergent soluble fraction from (Figure 1D) using L8C4 and 2 x 10^7 cell equivalents per lane. Coomassie blue stained gel was used as a loading control. (C) Proteasome inhibitor MG132 was added to parental, Tb927.10.8870-/- and Tb927.10.8870 add back cell lines for 8 hours. Western blot was performed on whole cell lysates of parental, Tb927.10.8870-/- and Tb927.10.8870 add back cell lines using monoclonal antibody L8C4 that recognizes PFR2. Coomassie blue stained gel and mAb25 was used as a loading control. The experiment was repeated twice and a representative blot is shown. (D) Graph and table showing the relative expression of PFR2 for the parental, null mutant and add back cells after MG132 treatment. PFR2 signal relative to the mAb25 signal was determined and then normalised to the parental untreated sample for each experiment.

A

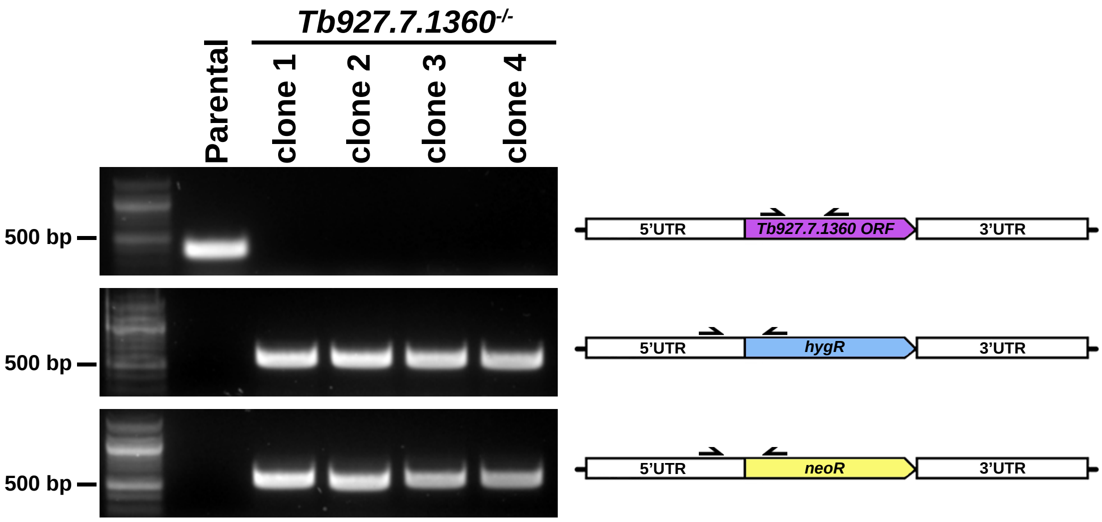

B

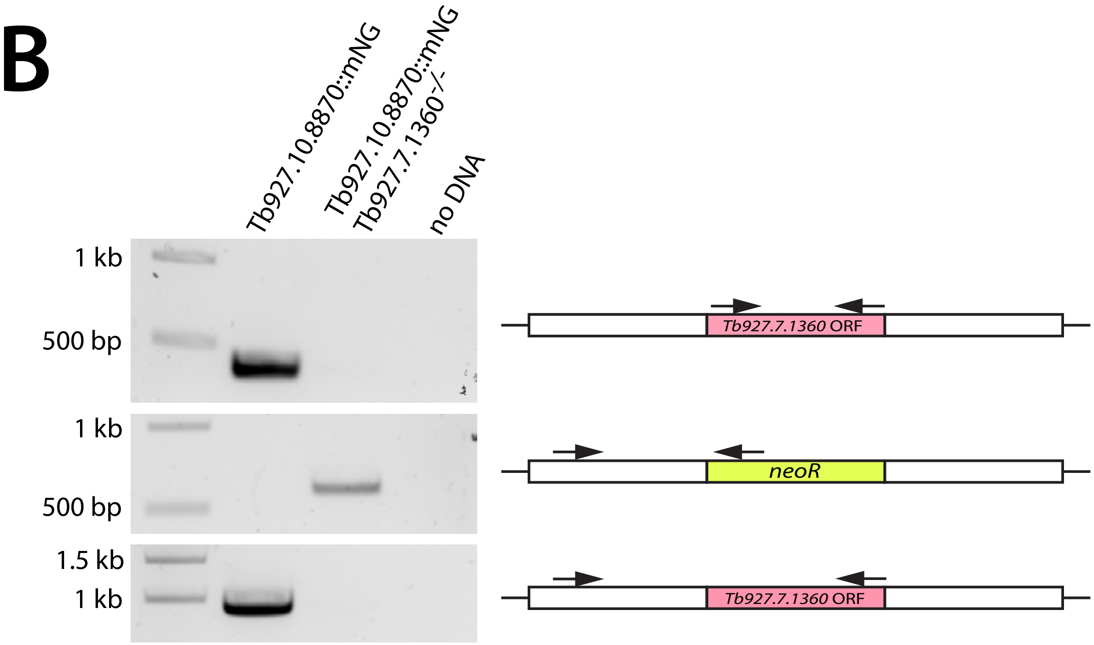

Figure S3. (A) Schematic and PCR confirmation of Tb927.7.1360 gene deletion. gDNA from 4 Tb927.7.1360-/- mutant clones and the parental cells was analysed by PCR. PCR confirmed that Tb927.7.1360 ORF was no longer present in the null mutant clones and that the resistance markers had integrated correctly. Tb927.7.1360-/- clone 1 was used for all subsequent experiments. (B) Schematic and PCR confirmation of Tb927.7.1360 gene deletion from cell line expressing Tb927.10.8870::mNG. gDNA from 1 mutant clone and the parental cells was analysed by PCR. PCR confirmed that Tb927.7.1360 ORF was no longer present.

Journal of Cell Science • Supplementary information
